# Applicability of Supraclavicular Oxygenated and Total Hemoglobin Evaluated by Near-Infrared Time-Resolved Spectroscopy as Indicators of Brown Adipose Tissue Density in Humans

**DOI:** 10.3390/ijms20092214

**Published:** 2019-05-06

**Authors:** Shinsuke Nirengi, Sayuri Fuse, Shiho Amagasa, Toshiyuki Homma, Ryotaro Kime, Miyuki Kuroiwa, Tasuki Endo, Naoki Sakane, Mami Matsushita, Masayuki Saito, Yuko Kurosawa, Takafumi Hamaoka

**Affiliations:** 1 Division of Preventive Medicine, National Hospital Organization Kyoto Medical Center, Clinical Research Institute, Kyoto 612-8555, Japan; shi.nirengi@gmail.com (S.N.); nsakane@gf6.so-net.ne.jp (N.S.); 2 Department of Sports Medicine for Health Promotion, Tokyo Medical University, Tokyo 160-8402, Japan; fuse@tokyo-med.ac.jp (S.F.); kime@tokyo-med.ac.jp (R.K.); mkuroiwa@tokyo-med.ac.jp (M.K.); endo-tasuki-fv@ynu.jp (T.E.); yuko.kurosawa.2011@gmail.com (Y.K.); 3 Department of Preventive Medicine and Public Health, Tokyo Medical University, Tokyo 160-8402, Japan; shiho.ama@gmail.com; 4 Faculty of Sports and Health Science, Daito Bunka University, Higashimatsuyama-shi, Saitama 355-8501, Japan; t-homma@ic.daito.ac.jp; 5 Department of Nutrition, Tenshi College, Sapporo 065-0013, Japan; matsushita@tenshi.ac.jp; 6 Hokkaido University, Sapporo 060-0818, Japan; ms-consa@krf.biglobe.ne.jp

**Keywords:** ^18^F-fluorodeoxyglucose-positron emission tomography, noninvasive, brown adipose tissue (BAT), seasonal temperature fluctuations, cold-induced thermogenesis, thermogenic functional ingredients

## Abstract

Brown adipose tissue (BAT) may potentially be used in strategies for preventing lifestyle-related diseases. We examine evidence that near-infrared time-resolved spectroscopy (NIR_TRS_) is capable of estimating human BAT density (BAT-d). The parameters examined in this study are total hemoglobin [total-Hb]_sup_, oxygenated Hb [oxy-Hb]_sup_, deoxygenated Hb [deoxy-Hb]_sup_, Hb O_2_ saturation (StO_2sup_), and the reduced scattering coefficient in the supraclavicular region (μ_s_’_sup_), where BAT deposits can be located; corresponding parameters in the control deltoid region are obtained as controls. Among the NIR_TRS_ parameters, [total-Hb]_sup_ and [oxy-Hb]_sup_ show region-specific increases in winter, compared to summer. Further, [total-Hb]_sup_ and [oxy-Hb]_sup_ are correlated with cold-induced thermogenesis in the supraclavicular region. We conclude that NIR_TRS_-determined [total-Hb]_sup_ and [oxy-Hb]_sup_ are useful parameters for evaluating BAT-d in a simple, rapid, non-invasive manner.

## 1. Introduction

Human brown adipose tissue (BAT) functions as a tissue for non-shivering thermogenesis in response to cold exposure, and it has been shown to be present in larger amounts in winter [1,2]. It is reported that human BAT is related to lower body weight [1,2] and enhanced glucose tolerance [3]. Daily cold exposure has been shown to increase BAT activity, not only in healthy subjects [1,4,5] but also in obese individuals [6] and patients with type 2 diabetes [7]. Thus, BAT is expected to be utilized in strategies for preventing or treating obesity and lifestyle-related diseases such as diabetes.

It is generally accepted that cold-induced activation of human BAT can be detected by ^18^F-fluorodeoxyglucose (FDG)-positron emission tomography (PET), used in conjunction with computed tomography (CT) (^18^FDG-PET/CT) [4,5,6,7]. However, ^18^FDG–PET/CT has several limitations, including enormous instrumentation costs, ionizing radiation exposure, and acute cold exposure [8]. These make repeated ^18^FDG–PET/CT measurements difficult, and hinder human studies, specifically longitudinal ones. Thus, a noninvasive, simple method, that does not require exposure to cold and/or ionizing radiation, is desirable. 

Near-infrared time-resolved spectroscopy (NIR_TRS_) is considered to be a noninvasive alternative method for evaluating BAT activity. Uniquely among commercially-available NIR techniques, NIR_TRS_ can evaluate optical properties, such as the absorption coefficient (μ_a_) and reduced scattering coefficient (μ_s_’), and therefore it can be used to noninvasively quantify tissue oxygenated hemoglobin [oxy-Hb], deoxygenated Hb [deoxy-Hb], and total Hb [total-Hb] concentrations. Among these NIR_TRS_ parameters, [total-Hb] and μ_s_’ have been investigated for evaluating BAT, as potential indices of blood volume (or tissue vasculature) and mitochondrial concentration [9], respectively. Hence, we postulated that, as BAT has abundant capillaries and mitochondria, NIR_TRS_ should allow assessment of vascular or mitochondrial density in BAT (BAT-d) in the supraclavicular region by measurement of [total-Hb]_sup_ and µ_s_′_sup_. It was reported that [total-Hb]_sup_ and µ_s_′_sup_, as determined by NIR_TRS_ under both thermoneutral and cold conditions, is positively correlated with cold-induced ^18^FDG–PET/CT parameters in the supraclavicular region, which potentially contains BAT deposits, but not in the deltoid muscle region control site [10]. Further, a longitudinal study reported that [total-Hb]_sup_ increases with the FDG-PET/CT parameter during repeated thermogenic capsinoid intake [11], which is known to increase BAT activity and mass. NIR_TRS_ parameters, in particular [total-Hb]_sup_, can therefore evaluate BAT density under thermoneutral conditions.

However, there is a lack of reliable data on the variation of NIR_TRS_ parameters (not only [total-Hb]_sup_ and µ_s_′_sup_, but also other parameters) with seasonal changes and during acute cold-induced thermogenesis (CIT), both of which correlate with increased BAT activity. Thus, the purpose of this study is to examine the ability of NIR_TRS_ measurement in the supraclavicular region to evaluate BAT-d in humans. The evidence we present comprises differences in NIR_TRS_ parameters measured in summer and winter, as well as correlations between the NIR_TRS_ parameters and CIT.

## 2. Results

**Figure 1:**
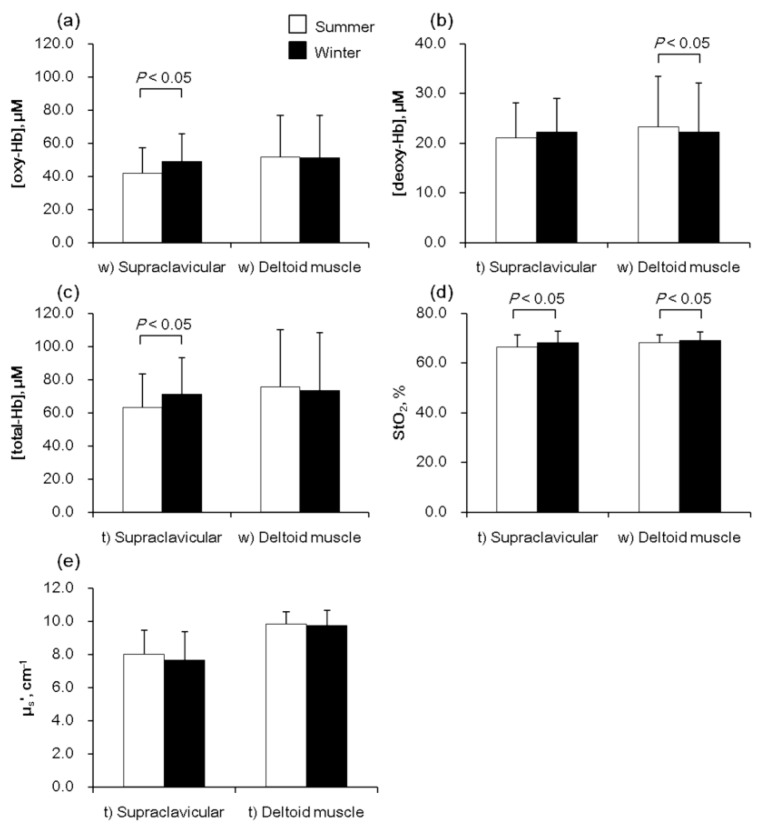
Near-infrared time-resolved spectroscopy results for 58 subjects measured during two seasons. (**a**) Oxygenated hemoglobin concentration [oxy-Hb], (**b**) deoxygenated Hb concentration [deoxy-Hb], (**c**) total Hb concentration [total-Hb], (**d**) Hb O_2_ saturation (StO_2_), and (**e**) reduced scattering coefficient (µ_s_’) of 760 nm in the supraclavicular region, and as a reference, in the deltoid region. Results are presented as mean values and the error bars indicate standard deviations. ^t)^ Paired *t*-tests or ^w)^ Wilcoxon signed-rank tests were performed to determine the significance of seasonal differences.

We found that [oxy-Hb]_sup_ (42.0 ± 14.9 vs. 48.9 ± 16.7 μM, *p* < 0.01) and [total-Hb]_sup_ (63.1 ± 20.6 vs. 71.2 ± 22.1 μM, *p* < 0.01) increased significantly, by 16.5% and 12.8%, respectively, from summer to winter, while there were no significant changes to the corresponding parameters for the deltoid muscle region ([oxy-Hb]_del_ and [total-Hb]_del_) that served as a control site. The winter measurement of [deoxy-Hb]_del_ was slightly but significantly greater, by only 4.5%, than the value in summer. StO_2sup_ and StO_2del_ were found to be higher in winter, compared to summer, in both the supraclavicular and deltoid muscle regions (*p* < 0.05) (Figure 1). The values of [total-Hb]_sup_ and [oxy-Hb]_sup_ showed very high correlations in both summer (*r* = 0.97, *p* < 0.01) and winter (*r* = 0.98, *p* < 0.01). The BAT-positive rate, defined as a [total-Hb]_sup_ value greater than 74.0 µM [10], was found to be 48.3% in winter, which is significantly higher than the value obtained from the measurement in summer (27.6%) (*p* < 0.05). 

Table 1 reveals the relationships between NIR_TRS_ parameters at 19 and 27 °C and CIT in winter. We found a significant correlation between [total-Hb]_sup_ (*r* = 0.48–0.64, *p* < 0.01) or [oxy-Hb]_sup_ (*r* = 0.49–0.62, *p* < 0.05) and CIT, both at 27 and 19 °C. However, the correlation between [deoxy-Hb]_sup_ and CIT is significant only at 27 °C. StO_2sup_ and μ_s_’_sup_ are not significantly correlated with CIT at 19 or 27 °C. None of the parameters for the deltoid region are significantly correlated with CIT.

## 3. Discussion

We found a significant increase only in values for [oxy-Hb]_sup_ and [total-Hb]_sup_ without any changes in the corresponding parameters for the control region, indicating that [oxy-Hb]_sup_, but not [deoxy-Hb]_sup_, has an accuracy similar to that of [total-Hb]_sup_ for detecting seasonal variations in BAT-d. Furthermore, although the reason is unclear, the decrease in [deoxy-Hb] in the deltoid region in winter indicates either that muscle metabolism decreased in the same period or that [deoxy-Hb] is a less stable index than [oxy-Hb] or [total-Hb]. We also found a significant correlation between CIT and each of the parameters [total-Hb]_sup_, [oxy-Hb]_sup_, and [deoxy-Hb]_sup_ at 27 °C, but not between CIT and any other parameters. Taken together, among the indicators determined by NIR_TRS_, [total-Hb]_sup_, and [oxy-Hb]_sup_ provide a reliable, sensitive, and specific means of evaluating BAT-d in both cross-sectional and longitudinal studies.

Recently a study aimed at investigating the association between near-infrared continuous-wave spectroscopy (NIR_CWS_) parameters (StO_2_, total-Hb, oxy-Hb, and deoxy-Hb), and BAT volume and activity estimated by ^18^FDG–PET/CT, has been reported [12]. The experiments were conducted by following the current cold exposure recommendations, and the measurements were obtained in the supraclavicular and forearm regions in young healthy women. No association between the NIR_CWS_ parameters and BAT volume and activity under warm conditions was observed in this study. Similarly, cold-induced changes in the NIR_CWS_ parameters were not found to be associated with BAT volume and activity. NIR_CWS_, therefore, does not seem to be a valid technique for indirectly assessing BAT in young healthy women. Moreover, another previous study examined the relationship between NIR_CWS_ and ^18^FDG-PET/CT parameters in subjects with low and high levels of BAT during cold exposure [13]. The adjusted supraclavicular StO_2_ (adjStO_2_) parameter, which is relative to the value in a control area (deltoid region), measured by NIR_CWS_, was significantly correlated with oxygen uptake by BAT in the high BAT group. 

The most common, commercially-available, near-infrared spectroscopy (NIRS) modality is NIR_CWS_. However, NIR_CWS_ only provides relative values of tissue oxygenation. Further, the depth of light penetration is approximately 15 mm for a 30-mm optode separation in NIR_CWS_ [14]. Thus, the main reason for the inability of the method to provide quantitative data, is that the path of NIR light through biological tissues is undetermined [14,15,16]. In contrast, NIR_TRS_ is a method employing picosecond light pulse emissions from the surface of the skin to measure the time distribution of photons scattered and/or absorbed in tissue that is several centimeters distant from the point of light emission. It noninvasively quantifies a range of tissue optical properties, including μ_a_, μ_s_′, and light path length, allowing calculation of tissue [oxy-Hb], [deoxy-Hb], [total-Hb], and StO_2_ [16,17,18]. It can also provide absolute values for tissue hemodynamics. Furthermore, according to a recent study [19], the mean depth of light penetration should be greater (approximately 20 mm at the same 30-mm optode separation), and more homogeneous in NIR_TRS_. Thus, it is clear that instrumentation differences between NIR_CWS_ and NIR_TRS_ influence the accuracy of these methods with respect to BAT detection.

As BAT is activated by acute cold exposure, it is believed that it increases in winter, and this was confirmed by the majority of the ^18^FDG–PET/CT studies [2,20,21], with the sole exception being a paper showing that BAT activity was higher in early winter than in late winter or early spring [22]. In one cross-sectional study, [total-Hb]_sup_ was reported to be significantly higher in winter than in summer [23]. Here, we confirm that [total-Hb]_sup,_ [oxy-Hb]_sup_, and StO_2sup_ increase in winter, which is consistent with the increase in BAT activity determined via the ^18^FDG-PET/CT studies [2,20,21,22]. To examine how BAT-d could be evaluated from data collected in summer, we correlated winter and summer data. There was a significant correlation between the summer and winter BAT indicators measured in the supraclavicular region, including [total-Hb]_sup_ (*r* = 0.78, *p* < 0.01), [oxy-Hb]_sup_ (*r* = 0.77, *p* < 0.01), and µ_s_’_sup_ (*r* = 0.52, *p* < 0.01). Thus, it could be possible to use these parameters determined in summer as an alternative to the winter parameters, which data might be unavailable, albeit acknowledging the lower accuracy of the summer data. Although we cannot identify the reason why [deoxy-Hb]_del_ decreased and StO_2del_ increased in winter, muscle vascularity and metabolism might be expected to show seasonal changes, and this should be further elucidated in a future study.

It is well known that whole-body oxygen consumption increases under non-shivering cold conditions, and that the mechanism involved comprises CIT brought about by the upregulation of uncoupling protein-1 in brown adipocytes. Those who have significantly greater BAT activity exhibit larger CIT than those with lower BAT activity. In addition, there is a correlation between BAT activity and CIT [1,24]. We have previously observed a strong correlation between [total-Hb]_sup_ or µ_s_′_sup_ and mean standardized uptake value (SUV_mean_) assessed by ^18^FDG–PET/CT [10]. We have demonstrated in this study that [total-Hb]_sup_ and [oxy-Hb]_sup_ might be reliable markers for evaluating BAT activity. However, we also showed that StO_2sup_ and µ_s_′_sup_ are less sensitive as markers, which is in contrast with the results of previous studies that showed a significant correlation between adjStO_2_ or µ_s_′ and ^18^FDG–PET/CT parameters [10,12].

For *in vivo* imaging, the short-wavelength infrared region (SWIR; 1000-2000 nm) provides several advantages over the visible and near-infrared regions: In blood and tissue, there is a general lack of autofluorescence, low light absorption, and reduced scattering upon irradiation with these wavelengths. A recent animal study using SWIR with contrast agents has demonstrated visualization of BAT characteristics [25]. However, this imaging protocol is not suitable for human subjects because of the necessity of injecting contrast agents. Further, light absorption by water limits photon penetration through biological tissue when wavelengths above 900 or 1000 nm are used for emission or detection [17], as was reported for the aforementioned BAT visualization experiment. To allow better penetration, almost all human NIRS studies, in common with our work, have utilized wavelengths in the region of 650–850 nm [10,11,12,13,14,15,16,17,18,19]. 

Our study has several limitations that we now discuss. First, the supraclavicular region is heterogeneous, and hence any NIR_TRS_ index results might be influenced by multiple tissue types other than BAT (e.g., white adipose tissue, muscle tissue, etc.). However, our previous work revealed a significant relationship between [total-Hb] and the ^18^FDG-PET/CT index in the supraclavicular region (*r* = 0.74), but not in the deltoid muscle region. Second, NIR_TRS_ cannot differentiate Hb from myoglobin (Mb), and it has been reported that Mb is present in BAT [26]. In order to investigate the proportion of Hb in human BAT, ^1^proton (^1^H)-magnetic resonance spectroscopy measurements are needed. Third, NIR_TRS_ indices could be interpreted as measurements of BAT density rather than BAT activity. Therefore, we believe that NIR_TRS_ does not detect the oxidative metabolism of BAT in either winter or summer. Fourth, the supraclavicular region is one of the representative locations for BAT deposits. We examined this location, because NIRS can only be applied with a mean light depth of 20 mm at a 3-cm optode separation [19]; unfortunately, we were not able to evaluate other deeper regions in which BAT is deposited. 

## 4. Materials and Methods

### 4.1. Subjects and Study Design

We tested whether near-infrared time-resolved spectroscopy (NIR_TRS_) parameters differ between seasons, taking measurements in summer [minimum temperature, 23.2 (22.0, 25.8) °C; maximum temperature, 32.3 (28.4, 33.7) °C, median (the first quartile, the third quartile)] and winter [minimum temperature, 3.2 (0.5, 5.4) °C; maximum temperature, 12.2 (9.6, 16.1) °C]. We recruited 58 subjects [men/women, 35/23; age, 40.5 (25.8, 47.0) year; BMI, 21.8 (20.4, 23.8) kg/m^2^, median (the first quartile, the third quartile)] to investigate changes in NIR_TRS_ measurements conducted in the range of 23 to 27 °C.

We also investigated whether or not NIR_TRS_ parameters were correlated with cold-induced thermogenesis (CIT), in 18 young men [age, 20.0 (19.0, 21.0) year; BMI, 24.2 (21.6, 25.7) kg/m^2^]. After fasting for 6–12 h, the subjects rested in an air-conditioned room at 27 °C, wearing light clothing (usually a T-shirt with underwear) for 20 min. This was followed by 5 min of NIR_TRS_ measurements. Then, the subjects entered an air-conditioned room at 19 °C and rested for 2 h; they also intermittently placed their feet on an ice block wrapped in cloth, usually for 4 min every 5 min [1,2]. The NIR_TRS_ parameters and energy expenditure were evaluated before and during the 2-h cold exposure.

All subjects gave informed consent for their participation in the study before the experiments were carried out. The study was conducted in accordance with the Declaration of Helsinki, and the protocol was approved by the Ethics Committees of Ritsumeikan University (IRB 2014-022, 8 December, 2014) and Tokyo Medical University (IRB 2017-199, 26 December, 2017).

### 4.2. NIR_TRS_ Parameters

The NIR_TRS_ parameters were measured by using a commercial NIR_TRS_ device (TRS-20; Hamamatsu Photonics K.K., Hamamatsu, Japan). Each measurement procedure required 1 min. The probes were placed on the skin of the supraclavicular region that potentially contained brown adipose tissue (BAT), and on the deltoid muscle region as a control site. The participants were required to remain in a sitting position during the measurements, as previously described [10,11,21]. Compared to visible light wavelengths, NIR wavelengths (700 to 3000 nm) are scattered less and, consequently, they show better penetration in biological tissue. However, light absorption by water limits tissue penetration above a limiting wavelength of about 900 nm; thus, the 650–850-nm range is suitable for measurements [27]. Accordingly, we used NIR wavelengths of 760, 800, and 830 nm to evaluate [oxy-Hb], [deoxy-Hb], and [total-Hb], respectively. With the 3 cm probe used in this study, light can penetrate to a mean depth of 2 cm [19], where BAT is potentially located. For the sake of accuracy, it should be noted that NIR_TRS_ cannot distinguish Hb from myoglobin (Mb). Hence, many studies have been reported in which results are presented as oxy-Hb/oxy-Mb, deoxy-Hb/deoxy-Mb, and total-Hb/total-Mb concentrations [17]. However, for simplicity, in this work we present the absorption measurements as [oxy-Hb], [deoxy-Hb], and [total-Hb].

Tissue was illuminated via a 200-μm-core-diameter optical fiber by a tunable picosecond pulsed light source (100-ps full width at half-maximum; 5-MHz repetition rate; 80-μW average power for each wavelength). The emitted photons penetrated the tissue, and were reflected into a 3-mm-diameter optical bundle fiber, through which they were sent to a photomultiplier tube for single-photon detection, using a signal processing circuit for time-resolved measurements. The nonlinear least-squares method was used to fit the digitized temporal profile measurement data with a theoretical temporal profile; the latter was derived from the analytical solution of the photon diffusion theory with a semi-infinite homogeneous reflectance model. After convolution with the instrumental response function to compensate for the time response of the instrument itself, the absorption and scattering coefficients, µ_a_ and μ_s_′, at 760, 800, and 830 nm were obtained using the least-squares fitting method. Then, absolute [total-Hb], [oxy-Hb], [deoxy-Hb], and StO_2_ values were calculated [17,28].

### 4.3. Cold Induced Thermogenesis (CIT)

Whole-body energy expenditure was estimated in winter using an automatic respiratory gas analyzer (AE300S, Minato Medical Science Co., Ltd., Tokyo, Japan) before (at 27 °C) and during 2 h of cold exposure (at 19 °C), during which the participants wore light clothing. Stable values for the 10-min period at the end of the 2-h duration of cold exposure were used as the measured values of energy expenditure. Thus, CIT values were calculated as differences between energy expenditure at 27 °C and at the end of the period of cold exposure at 19 °C [1,24].

### 4.4. Statistical Analyses

Data are expressed as median (first quartile, third quartile) or mean ± standard deviation (SD). If a normal distribution was detected by the Shapiro-Wilk test, we used a paired *t*-test to test for seasonal differences in the NIR_TRS_ parameters; otherwise, a Wilcoxon signed-rank test was conducted. The Pearson product-moment correlation coefficient was evaluated to analyze the relationship between CIT and NIR_TRS_ parameters at 19 and 27 °C. BAT-positive rates were compared in winter and summer using chi-squared tests. All analyses were performed using SPSS software (IBM SPSS Statistics 25, IBM Japan, Tokyo, Japan) and *p* < 0.05 values were considered statistically significant.

## 5. Conclusions

We observed that seasonal temperature fluctuations influenced [total-Hb]_sup_ and [oxy-Hb]_sup_ and that there were significant correlations between CIT and each of the parameters [total-Hb]_sup_, [oxy-Hb]_sup_, and [deoxy-Hb]_sup_ (only at 27 °C) in winter. These relationships were seen specifically in measurements of the supraclavicular region, which potentially contains BAT deposits. Thus, among the indicators determined by NIR_TRS_, [total-Hb]_sup_ and [oxy-Hb]_sup_ provide a reliable and sensitive means for evaluating BAT-d in both cross-sectional and longitudinal studies. We conclude that NIR_TRS_ is a new approach for evaluating BAT-d, and could prove to be a useful tool for simple, non-invasive measurements in the clinic. However, a further validation study is definitely needed to confirm our results via side-by-side NIR_TRS_ and conventional gold-standard ^18^FDG–PET/CT measurements.

## Figures and Tables

**Table 1:** Relationship between near-infrared time-resolved spectroscopy (NIR_TRS_) parameters at 27 °C or 19 °C and cold-induced thermogenesis (CIT) *^a^*.

		Supraclavicular Region	Deltoid Muscle Region
[total-Hb]	27 °C	0.64 ^※^	0.24
	19 °C	0.48 ^※^	0.21
[oxy-Hb]	27 °C	0.62 ^※^	0.25
	19 °C	0.49 ^※^	0.15
[deoxy-Hb]	27 °C	0.70 ^※^	0.20
	19 °C	0.40	0.30
StO_2_	27 °C	−0.38	0.20
	19 °C	−0.11	−0.24
μ_s_’	27 °C	−0.02	0.30
	19 °C	0.08	0.39

Determined as averages of near-infrared time-resolved spectroscopy and cold-induced thermogenesis (CIT), results for 18 young lean men in an air-conditioned room at 27 or 19 °C. ^※^ *p* < 0.05.

## Abbreviations

adjStO_2_	adjusted supraclavicular hemoglobin oxygen saturation
BAT	brown adipose tissue
BAT-d	vascular or mitochondrial density in brown adipose tissue
CIT	cold-induced thermogenesis
CT	computed tomography
deoxy-Hb	deoxygenated hemoglobin
FDG	18F-fluorodeoxyglucose
Hb	hemoglobin
NIRS	near-infrared spectroscopy
NIR_CWS_	near-infrared continuous-wave spectroscopy
NIR_TRS_	near-infrared time-resolved spectroscopy
oxy-Hb	oxygenated hemoglobin
PET	positron emission tomography
StO_2_	hemoglobin oxygen saturation
SUV_mean_	mean standardized uptake value
total-Hb	total hemoglobin
µ_a_	absorption coefficient
µ_s_’	scattering coefficient
